# Vacuolar ATPase as a potential therapeutic target and mediator of treatment resistance in cancer

**DOI:** 10.1002/cam4.1594

**Published:** 2018-06-21

**Authors:** Bradleigh Whitton, Haruko Okamoto, Graham Packham, Simon J. Crabb

**Affiliations:** ^1^ Southampton Cancer Research UK Centre University of Southampton Southampton UK; ^2^ Biological Sciences Faculty of Natural and Environmental Sciences University of Southampton Southampton UK

**Keywords:** cancer, drug resistance, invasion, metastasis, novel therapy, V‐ATPase

## Abstract

Vacuolar ATPase (V‐ATPase) is an ATP‐dependent H^+^‐transporter that pumps protons across intracellular and plasma membranes. It consists of a large multi‐subunit protein complex and influences a wide range of cellular processes. This review focuses on emerging evidence for the roles for V‐ATPase in cancer. This includes how V‐ATPase dysregulation contributes to cancer growth, metastasis, invasion and proliferation, and the potential link between V‐ATPase and the development of drug resistance.

## 1 INTRODUCTION

V‐ATPase is a multi‐protein complex that catalyzes the ATP‐dependent transport of protons across intracellular and plasma membranes. The resulting acidification of organelle lumens and the extracellular space influences a diverse range of cellular processes, many of which are dysregulated in cancers. Here, we review current evidence that V‐ATPase function promotes multiple cancer‐associated hallmarks, especially invasion and metastasis, and that V‐ATPase inhibition may have utility as a novel anti‐cancer therapeutic strategy.

## 2 V‐ATPASE STRUCTURE AND FUNCTION IN NORMAL PHYSIOLOGY

**Figure 1:**
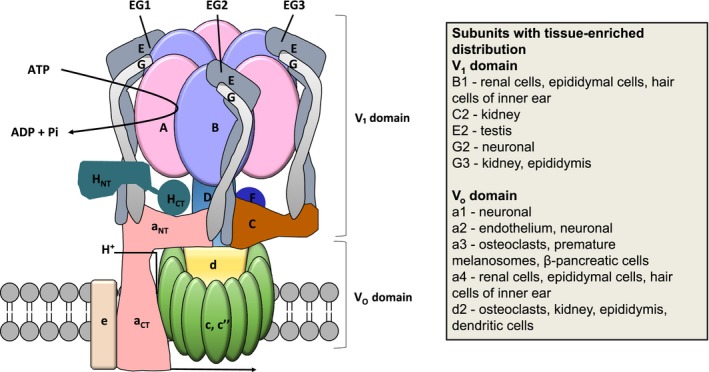
V‐ATPase structure. The V_1_ domain contains the A_3_B_3_ catalytic hexamer, the peripheral stalk made up of subunits E, G, C, and H, and D and F of the central rotor. ATP hydrolysis occurs in the A_3_B_3_ catalytic hexamer and the energy generated is used to drive the rotary mechanism. The V_o_ domain is integrated into the membrane and is responsible for proton translocation; it consists of subunits a, d, e and the proteolipid ring made up of c and c″. Many of the subunits are expressed in the form of multiple isoforms; the tissue enriched localization of some of the important isoforms is shown

Mammalian V‐ATPase comprises 13 distinct subunits that are part of either the cytosolic V_1_ (A, B, C, D, E, F, G and H) or membrane‐integral V_o_ (a, c, c″, d and e) domains. ATP hydrolysis occurs within the core A_3_B_3_ hexamer of the V_1_‐domain and drives rotation of the central stalk resulting in H^+^‐transport via the V_o_‐domain. The complex is orientated such that protons are transported from the cytoplasm to the lumen of organelles or, for plasma membrane‐located V‐ATPase, to the extracellular space. The key structural and functional features of V‐ATPase are summarized in Figure 1, and readers are referred to recent reviews for more detailed information.^1^, ^2^ To clarify, the nomenclature of the genes encoding the subunits A in the V_1_ and a in V_o_ domains are ATP6V1A and ATP6V0A, respectively.

The peripheral stalks in the V_1_ domain are composed of 3 V_1_E and V_1_G subunits, which form dimers that are connected by the V_1_C and V_1_H subunits and the N‐terminus of V_o_a in the V_o_ domain (Figure 1). The peripheral stator has an important role in tethering the A_3_B_3_ hexamer to the N‐terminus of subunit V_o_a, thus resisting the torque generated by the rotation of the central stalk. Protons enter the cytoplasmic hemi‐channel in the V_o_ domain via the transmembrane V_o_a subunit and bind to a conserved glutamic acid residue in the V_o_c or V_o_c″ subunit. The clockwise rotation of the central stalk sequentially rotates the proteolipid ring, allowing the protonated glutamic acid residues to come into contact with an arginine residue within a hemi‐channel in the V_o_a subunit. This results in the deprotonation of the glutamic acid residue and the release of the proton into the lumen.

**Figure 2:**
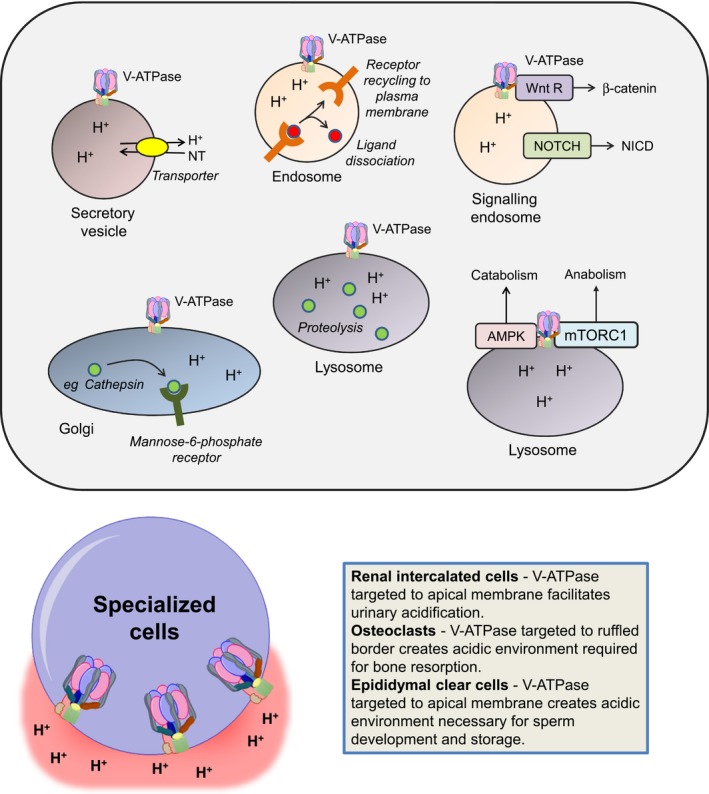
V‐ATPase function. A, Intracellular V‐ATPase regulates multiple intracellular processes. In secretory vesicles, V‐ATPase generates a proton gradient that is used to drive the H^+^‐dependent uptake of neurotransmitters. In endosomes, low pH promotes the dissociation of ligands, such as low density lipoproteins, from their receptors, facilitating receptor recycling to the plasma membrane. In signaling endosomes, V‐ATPase promotes signaling to β‐catenin downstream of Wnt receptors, and can promote NOTCH signaling via enhanced cleavage to generate NICD. In the Golgi, V‐ATPase promotes binding of hydrolases to the mannose‐6‐phosphate receptor which is important for their delivery to the lysosome. In lysosomes, low pH is required for optimal activity of acid‐dependent proteases, such as cathepsins. Lysosomal V‐ATPase also acts to coordinate activity of AMPK and mTORC1 to regulated cellular catabolism vs anabolism in response to shifting microenvironmental cues. Note the figure is designed to illustrate some key functions of V‐ATPase and is not intended to portray all of the diverse functions that have been ascribed to V‐ATPase. B, Plasma membrane functions of V‐ATPase in specialized cell types including renal intercalated cells, osteoclasts, and clear epididymal cells

Intracellular membrane‐associated V‐ATPase is ubiquitous in mammalian cells where it acidifies lysosomes, endosomes, and secretory vesicles, and thereby influences many processes associated with these organelles, including vesicular trafficking, endocytosis, autophagy, receptor recycling, and protein degradation.^1^ V‐ATPase‐dependent lysosome acidification also drives the coupled transport of small molecules/ions into the cytoplasm, especially of amino acids and calcium ions. A summary of some important V‐ATPase functions is shown in Figure 2.

Intracellular V‐ATPase plays important roles in several key cell signaling pathways.^2^, ^3^ For example, in some systems, V‐ATPase is required for optimal signaling downstream of NOTCH receptors as V‐ATPase‐mediated acidification of endosomes enhances γ‐secretase‐mediated proteolysis of NOTCH to generate the active NICD cleavage product.^4^, ^5^ However, in other cell systems (ie, triple negative breast cancer cells) V‐ATPase appears to negatively regulate NOTCH signaling as chemical inhibition of V‐ATPase activity or RNAi‐mediated knockdown of the V_o_a2 subunit, results in increased NOTCH signaling associated with increased NICD expression.^6^ Consistent with this inhibitory effect, conditional deletion of V_o_a2 in mouse mammary glands results in reduced mammary gland development, associated with increased NOTCH (and TGFβ) signaling.^7^ V‐ATPase is also required for Wnt signaling, including in developing *Xenopus* embryos, in which the V‐ATPase accessory protein, ATP6AP2 (also known as prorenin receptor), acts as an adaptor between Wnt receptor complexes and V‐ATPase.^8^

V‐ATPase acts as common activator for both mTORC1 signaling, which is activated under conditions of nutrient abundance and growth factor stimulation to promote cell growth, and AMPK (AMP‐activated protein kinase) which leads to enhanced catabolic activity.^9^ Thus, V‐ATPase coordinates a molecular switch between anabolism and catabolism in response to shifting metabolic cues. Although AMPK is classically activated in response to rising AMP/ATP ratios, recent studies have demonstrated that V‐ATPase plays a role in AMP‐independent AMPK activation. Thus, binding of aldolase to V‐ATPase in the absence of the glycolytic intermediate fructose‐1,6‐bisphosphate results in AMPK activation.^10^, ^11^

In contrast to the ubiquitous role of V‐ATPase at intracellular membranes, function of plasma membrane V‐ATPase is enriched in specific cell types, including osteoclasts and epididymal clear cells (where it is important for extracellular acidification) and renal epithelial intercalated cells (where it is important for acid secretion). In these cells, trafficking of V‐ATPase to the plasma membrane is regulated via intracellular signaling. For example, enhanced “delivery” from V‐ATPase‐rich intracellular vesicles increases the density of the complex in the apical plasma membrane of renal intercalated cells in response to reduced plasma pH.^12^ Here, PKA‐mediated phosphorylation of V‐ATPase controls fusion and endocytosis of V‐ATPase‐containing vesicles.^13^, ^14^ Interestingly, the PKA‐mediated increase in apical V‐ATPase in epididymal clear cells and renal intercalated cells is antagonized by AMPK and the V_1_A subunit appears to be a shared target for modulation by PKA and AMPK‐mediated phosphorylation.^14^, ^15^ Additionally, the V_o_a subunits are important for targeting V‐ATPases to the plasma membrane of specialized acidifying cells. For example, plasma membrane localization of V‐ATPase in osteoclasts and epididymal cells is associated with expression of tissue‐enriched V_o_a3/a4 subunits.^16^, ^17^ Key tissue‐enriched subunit isoforms are shown in Figure 1.

In addition to regulated trafficking, reversible association of V_1_ and V_o_ domains plays an important role in controlling V‐ATPase function. Disassociation of V_1_/V_o_ domains is accompanied by release of “free” V_1_C subunit, which plays a central role in the reassembly process. Multiple stimuli promote V‐ATPase assembly in mammalian cells such as the presence of growth factors, amino acid starvation, and increased glucose concentration.^1^, ^18^ For example, it has been proposed that in the presence of growth factors, such as EGF, increased V‐ATPase assembly is crucial to generate sufficient levels of amino acids from lysosomal protein degradation for mTORC1 stimulation.^19^ Increased V‐ATPase assembly due to amino acid starvation may be due to increased intracellular levels of free amino acids as a result of increased protein degradation in the lysosome.^20^ PI3‐kinase‐dependent signaling appears to play a central role in inducing V‐ATPase assembly in response to increased glucose, but not amino‐acid deprivation.^20^, ^21^ V‐ATPase assembly is also promoted by interaction with aldolase or phosphofructokinase^22^, ^23^ and this may be important for AMPK activation.^10^

V‐ATPase function can also be modulated by incorporation of specific subunit isoforms, many of which are expressed in a tissue‐enriched pattern (eg, V_o_a3/a4) or by binding of auxiliary subunits such as ATP6AP1 (Ac45) and ATP6AP2.^24^ V‐ATPase function has also been demonstrated to be influenced by the transmembrane protein TM9SF4 and ceramide synthase (LASS2/TMSG1) although the molecular basis for these functional links remain only partially understood.^25^, ^26^

## 3 DYSREGULATION OF V‐ATPASE IN CANCER

The bulk of the evidence linking dysregulation of V‐ATPase to cancer derives from studies showing altered expression or subcellular localization of specific V‐ATPase subunits in malignant cells versus normal counterparts and/or correlations between variable subunit expression and clinical features of disease.

For example, analysis of the TCGA database revealed that amplification/overexpression of ATP6VC1 was observed in 34% of human breast cancers and was associated with a relatively poor outcome.^27^ In a separate study, expression of the V_1_C1 subunit, which bridges between the V_1_ and V_o_ domains, was detectable in all examined stages of Barrett's esophagus (a precursor lesion for ESCC associated with an ~0.3% annual risk of progression to adenocarcinoma) using immunohistochemistry (IHC), indicating that its increased expression may play a role early in the transformation in these tissues.^28^ Interestingly, immunofluorescence staining revealed that at least a proportion of V‐ATPase was localized to the plasma membrane in ESCC cells.^29^ Furthermore, a recent detailed study using IHC showed that the V_1_E1 subunit was overexpressed in esophageal squamous cell carcinoma (ESCC) compared to normal esophagus. Unusually high V_1_E1 subunit protein level in cancer cells was associated with tumor invasiveness and lymph node (LN) metastasis, and with reduced disease‐free and overall survival, including in early stage disease. Importantly, V_1_E1 expression was an independent predictor of outcome^30^ and potentially used as a marker of disease. A similar study has investigated the significance of the V_1_A subunit in gastric cancer using IHC. Again, increased V‐ATPase subunit expression was observed in cancer, compared to normal, and high tumor expression was associated with poor differentiation, lymph node metastasis, advanced stage and was an independent predictor of poor outcome.^31^

Other studies have described expression of V‐ATPase/specific subunits in pancreatic cancer,^32^, ^33^ nonsmall cell lung cancer,^34^ oral squamous cell carcinoma,^35^, ^36^ ovarian cancer,^37^ cervical cancer,^38^ breast cancer,^39^, ^40^ gliomas,^41^ and hepatocellular carcinoma.^42^ A summary of studies investigating clinical significance of V‐ATPase in cancer is provided in Table 1. It is important to note that most of these studies focused on individual subunits and lack information on localization, expression of other subunits, and/or analysis of the ATP hydrolysis and proton transport activities of V‐ATPase. Further investigation into whether altered gene expression and/or protein accumulation of one of the subunits of V‐ATPase lead to malfunction of V‐ATPase function in the primary human tumors remains an important area for future investigation.

**Table 1:** Evidence for V‐ATPase subunit dysregulation in patient tumor tissues

Cancer type	Subunits investigated	Techniques	Key evidence	References
Breast	V_o_a	RT‐qPCR, IHC	V_o_a3 mRNA expression was upregulated in all breast cancer tissues tested compared to normal tissue and a3 mRNA expression correlated with tumor grade. Invasive breast cancer tissue had greater a3 staining than ductal carcinoma in situ, suggesting a3 expression increases with invasive potential.	Cotter et al^39^
Breast and melanoma	V_o_a2	IHC, IF	Highly positive staining of V_o_a2 in breast and skin tumors compared to their respective normal tissues.	Katara et al^40^
Cervical	V_1_C1	IHC	V‐ATPase expression significantly increased in patients with cervical adenocarcinoma. In patients with bulky cervical tumor expression was correlated with poor disease‐free survival	Song et al^38^
Gastric	V_1_A	IHC	V_1_A overexpressed in gastric cancer tissue compared to normal tissue. V_1_A expression correlated with advanced tumor grade, vascular invasion, lymph node metastasis and was associated with worse survival than patients with negative V_1_A staining.	Liu et al^31^
Glioma	V_o_a	RT‐qPCR	V_o_a4 isoform increased compared to brain biopsies of epileptic patients.	Gleize et al^41^
Human hepatocellular carcinoma	V_o_c (ATP6L)	RT‐qPCR, WB, IHC	Greater ATP6L mRNA and protein expression in HCC tissues compared to normal liver tissue. Baf‐A1 inhibition retarded growth of HCC in liver of mice.	Xu et al^19^, ^42^
Lung	V‐ATPase complex	IHC	V‐ATPase complex overexpressed in NSCLC. Expression was significantly lower in grade II adenocarcinoma and squamous cell lung cancer than grade III. V‐ATPase expression was found to be positively correlated to drug resistance in NSCLC samples and a significant positive correlation was obtained for V‐ATPase expression and common cancer chemotherapeutic agents.	Lu et al^34^
Esophageal	V_o_a and V_1_C1	Confocal microscopy, IHC	V‐ATPase expressed in all stages of neoplastic progression in Barrett's esophagus.	Chueca et al^28^
	V‐ATPase complex	IHC/IF	Complex highly expressed in esophageal squamous cancer cells.	Huang et al^29^
	V_1_E1	IHC	V_1_E1 expression increased in esophageal cancer tissues than normal esophageal tissue and was directly correlated with tumor invasiveness and poor prognosis. Can act as independent prognostic factor.	Son et al^30^
Oral squamous cell carcinoma	V_1_C1	RT‐qPCR	V_1_C1 overexpressed in OSCC tissues compared to healthy oral mucosa samples.	Perez‐Sayans et al^36^
	V_1_C1	IHC	V_1_C1 overexpressed in OSCC tissues compared to healthy oral mucosa tissue.	Garcia‐Garcia et al^35^
Ovarian	V_o_a	IF, IHC	V_o_a2 expression increased and a3 subunits had greater staining in ovarian cancer tissues compared to normal ovarian tissues.	Kulshrestha et al^37^
Pancreatic	V_o_c	RT‐qPCR, IHC, IP	V_o_c overexpressed in pancreatic carcinoma tissues compared to normal pancreatic tissue. Expression was linked to invasive capabilities as the overexpression of V_o_c was characteristic of invasive ductal adenocarcinomas.	Ohta et al^32^
	V_1_E	IHC	V‐ATPase staining was significantly increased from low‐grade pancreatic intraepithelial neoplasia (PanIN) to invasive PDAC.	Chung et al^33^

IHC, Immunohistochemistry; IF, Immunofluorescence; WB, Western blot; IP, Immunoprecipitation.

An important new advance that moves beyond differences in subunit expression in cancer is the recent identification of V‐ATPase subunit mutations. Somatically acquired mutations of *ATP6V1B1* or the accessory subunit *ATP6AP1* are detected in ~20% of follicular lymphoma (FL).^43^, ^44^ These V‐ATPase mutations frequently co‐occur with mutations in the *RRAGC* gene encoding the Rag‐GTPase family member RagC which, with V‐ATPase and the V‐ATPase interaction partner Ragulator, form an amino acid “sensing” supercomplex which is required for mTORC1 activation.^45^ FL *RRAGC* mutations appear to be gain‐of‐function and promote inappropriate mTORC1 activity following amino‐acid depletion.^44^ Functional data are lacking, but V‐ATPase mutations may also promote inappropriate mTORC1 activation in FL. A relatively large number of V‐ATPase subunit sequence variants have been reported in the COSMIC database in a wide spectrum of cancer types^46^ suggesting that the occurrence and importance of V‐ATPase mutations may extend beyond lymphoma. However, validating studies will be required to confirm which sequence variants are truly cancer specific and have important functional consequences.

## 4 V‐ATPASE FUNCTION IN CANCER CELLS

Functional characterization of V‐ATPase in human cancer cells has focused on its role in potentiation of migration and invasion, consistent with the common association between elevated subunit expression and advanced stage/metastasis observed in several cancer types (Table 1). Increased cancer cell invasive activity is frequently associated with aberrant V‐ATPase plasma membrane localization suggesting that increased acidification of the extracellular space plays an important role.^29^, ^33^, ^34^, ^37^, ^47^, ^48^, ^49^, ^50^, ^51^, ^52^

Various cell lines have been used to study the functional links between V‐ATPase and invasive activity. For example, using 2 closely related human breast cancer cell lines, MCF10a and MCF10CA1a, Capecci et al found that the levels of mRNA encoding V_o_a1 and V_o_a3 were highest in the invasive MCF10CA1a cells.^48^ Furthermore, the V_o_a3 isoform was also expressed in lung and bone metastasis of B16‐F10 cells, suggesting this isoform might be relevant to the increased metastatic potential of melanoma cells.^51^ As previously discussed, V_o_a isoforms can be expressed in a tissue‐enriched pattern and are associated with plasma membrane localization. Therefore overexpression of these isoforms might be an example of a cancer cells hijacking a normal cellular process to increase H^+^ extrusion.

V‐ATPase in the invasive breast cancer MDA‐MB‐231 cell line was shown to be localized to the plasma membrane and V‐ATPase activity was significantly higher than less invasive cell lines. To investigate whether plasma membrane or intracellular V‐ATPases were responsible for the invasive phenotype of breast cancer cells, Cotter et al inhibited plasma membrane V‐ATPase activity in MDA‐MB‐231 cells with a monoclonal antibody to the V5 epitope of a V5 tagged V_o_c subunit construct (which, in contrast to pharmacological inhibitors or RNAi‐mediated subunit ablation, would be expected to specifically target plasma membrane localized V‐ATPase) and found that specific inhibition significantly decreased in vitro invasion.^49^ Other studies of breast cancer cells have also linked V‐ATPase subunit expression to invasion. For example, siRNA knockdown of V_o_a3 (but not a1, a2 or a4) significantly decreased invasion of MCF10CA1a cells. Interestingly, in this system, knockdown of V_o_a4 led to an increase in V_o_a3 expression and a combined knockdown of both V_o_a3 and a4 led to the greatest reduction in MCF10CA1a cell invasion.^48^

The precise mechanisms by which V‐ATPase promotes invasion/migration require further investigation, but appear multifactorial. V‐ATPase can increase the activity of extracellular proteases, such as cathepsins and matrix metalloproteinases, via both increased protease secretion and decreased extracellular pH which enhances activity of these enzymes.^53^, ^54^, ^55^ Once activated, these proteases increase extracellular matrix degradation and facilitate migration of cancer cells. For example, in ovarian cancer cells, V_o_a2 was found to be localized in endosomes and inhibition showed a decrease in MMP‐9 and ‐2 activity.^37^ A study in hepatocellular carcinoma (HCC) found that inhibiting the V_o_c subunit led to decreased MMP‐2 expression and extracellular MMP‐2 activity.^56^

Other potential mechanisms may involve modulation of intracellular organelles. Intracellular V‐ATPase may play a role in tumor cell invasion by either assisting in the activation of lysosomal proteases or by activating cytosolic proteins which aid in trafficking these proteases to the surface of the cell.^57^, ^58^ V‐ATPase localizes with vesicles containing the small GTPase Rab27B, which promotes secretion of proinvasive growth factors and is associated with poor prognosis in breast cancer. V‐ATPase inhibition alters distribution and size of Rab27B vesicles, and reduces collagen type I invasion, cell cycle, and invasive growth in the chorioallantoic membrane assay. Importantly, V‐ATPase inhibition reduces the Rab27B dependent extracellular secretion of Hsp90α, which is a molecular chaperone required for MMP‐2 activation.^57^

The role of V‐ATPase in tumor migration has also been linked to tumor cell stiffness. Recent evidence suggests that a loss of cell stiffness is correlated with an increase in invasion and proliferation.^59^ For example, in HCC V‐ATPase inhibition was shown to increase cell stiffness due to depletion of cholesterol in the plasma membrane and decreased Ras signaling.^60^ However, due to the complexity of the tumor microenvironment, the impact of V‐ATPase on tumor stiffness is not clear. In breast cancer, in vivo deletion of the ATP6V0A2 gene encoding V_o_a2 subunit caused a reduction in breast tissue stiffness due to defective extracellular matrix glycosylation and altered Golgi morphology, resulting in an inflammatory and metastatic phenotype. Furthermore, normal breast tissue from patients who had reported lymph node metastasis (LNM) was analyzed using IHC and was found to have significantly lower V_o_a2 expression than those without LNM, indicating that low V_o_a2 expression is associated with metastatic disease.^61^

V‐ATPases may also promote increased cancer cell migration via interaction with the cytoskeleton. In breast cancer 4T1 cells, following lentiviral depletion of V_1_C1 subunit expression, the actin cytoskeletons lost their regular orientation resulting in decreased migratory and invasive activity, suggesting that V_1_C1 is able to regulate actin cytoskeleton rearrangements.^62^ Moreover, V‐ATPase inhibition is associated with F‐actin reorganization in PC‐3 prostate cancer cells.^63^ In addition, inhibition of V‐ATPase using archazolid A or V_o_c subunit siRNA in SKBR3 breast cancer cells altered the distribution and localization of EGFR and Rac‐1, which are associated with cellular migration.^64^

In addition to effects on migration/invasion linked to extracellular acidification, it has also been proposed that H^+^‐extrusion by V‐ATPase can counter intracellular acidification associated with altered metabolism.^1^ Thus, cancer cell growth is often associated with a reprogramming of metabolism towards aerobic glycolysis. This is termed the Warburg effect and is associated with tumor hypoxia as well as activation of oncogenes, such as MYC and RAS, and inactivation of tumor suppressors, such as p53.^65^, ^66^, ^67^ This results in production of lactic acid and H^+^, and plasma membrane V‐ATPase may be key under these conditions for maintaining intracellular pH homeostasis.^68^ In addition, via its action as a coordinator of regulators of opposing anabolic versus catabolic pathways (eg, mediated via mTORC1 or AMPK, respectively).^10^ V‐ATPase may support “metabolic plasticity” allowing tumor cells to adapt to distinct and shifting tumor microenvironments (eg, nutrient/O_2_ replete versus limited).

In prostate cancer cells, V‐ATPase co‐localized with prostate specific antigen (PSA; a secreted tumor marker that is regulated by the androgen receptor [AR]) in the Golgi and V‐ATPase inhibition resulted in a relocalization of PSA to lysosome‐like intracellular vesicles.^50^ Interestingly, in androgen‐sensitive LNCaP prostate cancer cells, V‐ATPase inhibition also reduced *PSA* mRNA expression, suggesting upstream inhibition of AR's transcription activating activity.^50^

Finally, V‐ATPase expression in cancer cells may influence immune cells to promote tumorigenesis indirectly. For example, a soluble cleavage fragment of V_o_a2 (a2NTD) has been shown to polarize macrophages toward a “tumor‐associated” or M2 macrophage phenotype.^40^, ^69^

## 5 V‐ATPASE AS A POTENTIAL CANCER THERAPEUTIC TARGET

V‐ATPase function is intimately involved in the control of multiple normal cellular processes. However, genetic manipulation experiments suggest the potential to target V‐ATPase function in cancer cells, with relatively little effect in normal cells, both in vitro and in vivo. This suggests that cancer cells may become particularly dependent on V‐ATPase complex function, or of specific subunits, to support cancer‐associated hallmarks, including growth and survival.

In the breast cancer cell lines MDA‐MB‐231, MCF‐7, and MDA‐MB‐435, shRNA‐mediated knockdown of the V_1_C1 subunit significantly inhibited cell proliferation, whereas in the untransformed C3H10T1/2 cell line, there was no effect on proliferation.^27^ In addition, 4T1 cells with 90% V_1_C1 depletion had significantly less metastatic potential and reduced osteolytic lesions.^27^ Alternatively, siRNA knockdown of the ATP6L (V_o_c) subunit in HCCLM3 hepatocellular carcinoma cells resulted in significantly reduced invasion, decreased MMP‐2 expression, reduced average xenograft size, and a dramatic reduction in intrahepatic metastases.^56^ In support of this, siRNA‐mediated knockdown of the V_o_c subunit led to a reduction in both SK‐N‐MC and A‐673 Ewing sarcoma cell number.^47^

In addition to these genetic experiments, chemical inhibitors of V‐ATPase, plecomacrolide bafilomycin‐A1 (baf‐A1), concanamycin‐A (con‐A), benzolactone enamides, and archazolid, have been used to understand consequence of V‐ATPase inhibition in vitro and in vivo. Both baf‐A1 and con‐A bind to the V_o_c subunit responsible for proton translocation and are highly specific V‐ATPase inhibitors with IC50 values in the nanomolar range. Benzolactone enamides have been extracted from marine organisms such as the sponge *Haliclona sp*. and the tunicate *Aplidium lobatum* as well as the gram negative bacterium *Psudomonas sp*. and the myxobacterium *Chondromyces sp*. Archazolid is one of the novel V‐ATPase inhibitors originally isolated from *Archangium gephyra*, which competes with con‐A for binding to the V_o_c subunit and also has IC50 values in the low nanomolar range.^70^

V‐ATPase inhibition has been shown to reduce cancer cell growth and induce apoptosis in a number of cell lines across a range of cancer types. Importantly, chemical inhibition appears to show selectivity for cancer cells compared to normal cells. For example, baf‐A1 inhibition resulted in significant reductions in hepatoblastoma cell growth compared to normal human hepatocytes.^71^ One of the benzolactone enamides and a derivative of salicylihalamide was shown to have a significantly synergistic effect on the viability of NCI‐H1155 lung cancer cells when applied with paclitaxel increasing the sensitivity of the latter 1000‐fold.^72^, ^73^ Additionally, archazolid had a significantly increased toxic effect in SKBR3 breast carcinoma cells compared to nontumor MCF10A cells.^74^

The biological mechanisms in which V‐ATPase inhibition induces cancer cell death are diverse and complex. V‐ATPase inhibition has been shown to result in increased reactive oxygen species (ROS) in cancer cells^75^, ^76^, ^77^ and HIF1α upregulation.^74^ Furthermore, V‐ATPase inhibition induces caspase‐dependent apoptosis in invasive tumor cells via the mitochondrial pathways.^74^, ^78^ Archazolid was also shown to induce cell cycle arrest in MDA‐MB‐231 cells and double‐strand breaks in all cell lines investigated.^79^ This evidence indicates that V‐ATPase inhibition can induce a cellular stress response, autophagy, and eventually apoptosis in tumor cells. However, it should be noted that the relationship between V‐ATPase and autophagy is complex particularly as the V‐ATPase has a well‐established role in activation of lysosomal acid hydrolases that mediate proteolysis during autophagy.^80^, ^81^, ^82^ Moreover, although V‐ATPase inhibition can result in increased autophagic markers in some settings,^74^ V‐ATPase is required for activation of noncanonical autophagy.^83^ In a very recent study, the autophagy‐related protein ATG5 was demonstrated to displace ATP6V1E1 from V‐ATPase, causing it to accumulate in exosomes.^84^ The function of autophagy in cancer is also complex; both pro‐survival and pro‐death rolls have been described, dependent on cellular context.^85^

There is also evidence for the stimulation of signaling pathways as MAPKs such as ERK, p38, and JNK were upregulated in response inhibition which might potentially provide a stimulus to apoptosis.^78^ This is in agreement with findings in breast cancer as ERK activation was shown to be increased as a pro‐survival mechanism in response to baf‐A1. Interestingly, inhibition of ERK using sorafenib augmented bafilomycin mediated cell death in tumor cells under hypoxic conditions.^86^

Furthermore, iron chelators deferoxamine and 3‐AP, showed a synergistic cytotoxic effect when used in combination with archazolid. It was therefore suggested that V‐ATPase induced cytotoxicity may primarily be due to disturbed iron receptor recycling, impairing the iron metabolism of tumor cells.^79^ Consistent with this, the expression of genes responsive to a decrease in iron was greatly increased in response to V‐ATPase inhibition. Iron was also inversely related to the cytotoxic hypersensitivity of cancer cells and therefore may act as a key determinant of cancer cell sensitivity to V‐ATPase inhibition.^87^

In addition to these in vitro studies, chemical V‐ATPase inhibition has recently been shown to be effective using in vivo models. For example, in a 4T1‐Luc mouse breast cancer xenograft model, archazolid treatment inhibited lung metastasis at a concentration (1 mg/kg i.v.) which did not cause signs of obvious toxicity.^64^ Similar results were obtained using baf‐A1, in which treatment led to the reduction of average tumor volume by 50% in MCF‐7 and MDA‐MB‐231 xenograft mouse models. Again, no toxic side effects were observed using baf‐A1 (1 mg/kg). Additionally, when combined with sorafenib, it was shown that V‐ATPase treatment could result in tumor regression in MDA‐MD‐231 xenograft mice.^86^ Another study demonstrated that growth of a HepG2 orthotopic HCC xenograft model in nude mice was retarded by baf‐A1.^42^

Finally, the potential “repurposing” of protein pump inhibitors (PPI), which are widely used for the treatment of gastroesophageal reflux and gastric ulcers, has garnered considerable interest.^88^ Although deployed as H^+^/K^+^‐ATPase inhibitors, PPI such as omeprazole and esomeprazole, also inhibit V‐ATPase and can increase sensitivity to chemotherapeutics in vitro and in vivo.^89^, ^90^ However, it should be noted that the concentration of PPI's required to inhibit V‐ATPase is considerably higher than for H^+^/K^+^‐ATPase.^91^

## 6 V‐ATPASE AS A MEDIATOR OF RESISTANCE FOR CONVENTIONAL CANCER THERAPIES

In addition to a role in cancer cell growth, survival, migration, and invasion, V‐ATPase dysregulation is also linked to therapy resistance. This may be explained, in part, to studies linking drug resistance in cancer cells, to reversal of the normal pH gradient between the cytoplasm and extracellular environment.^1^ Mechanistically, in some cases, lowering of extracellular pH protonates drugs leading to impaired cellular entry and/or vesicular trapping. Indeed, it remains unclear whether correlations between V‐ATPase (subunit) dysregulation and poor clinical outcome (Table 1) reflect an impact of V‐ATPase on cancer cell behavior per se vs a response to treatment.

In breast cancer, V‐ATPase inhibition was able to induce apoptosis in the trastuzumab resistant JIMT‐1 cells, impair HER2 signaling, and decrease HER2 surface expression. Moreover, treatment with archazolid significantly reduced the proportion of strongly HER2 positive cells and tumor growth in a JIMT‐1 cell xenograft.^92^ Chemical V‐ATPase inhibition was also shown to overcome Bcl‐xL‐ and Bcl‐2 mediated resistance in Ms‐1 cells and induce apoptosis. Baf‐A1 was able to suppress the mitochondrial protective function of Bcl‐xL and allow the taxol to decrease MMP levels.^93^ V‐ATPase expression was found to be higher in cisplatin resistant cells than other drug‐resistant cell lines. It has been shown that baf‐A1 and cisplatin had a synergistic effect on cell cytotoxicity, which was higher in cisplatin resistant cells than cisplatin sensitive.^94^

Again, a potential role for V‐ATPase in drug resistance has been demonstrated using siRNA ablation of specific subunits. You et al used siRNA targeting the ATP6L (V_o_c) subunit in human drug resistant MCF‐7/ADR breast cancer cells. The knockdown cells were more sensitive to chemotherapeutic agents such as doxorubicin and 5‐FU than control MCF‐7/ADR cells. ATP6L knockdown was associated with an increase of lysosomal pH (ie alkalinization) and increase caspase‐3/7 activity and PARP expression.^95^

## 7 ROLE OF V‐ATPASE MODULATORY PROTEINS IN CANCER

In addition to the core V‐ATPase subunits (Figure 1), proteins which directly modulate V‐ATPase activity have been shown to contribute to cancer progression. LASS2/TMSG1 is a negative V‐ATPase regulatory protein which directly binds to the V_o_c subunit and was found to be inversely related to the metastatic potential of tumor cells. It was shown to be expressed at low levels in the highly metastatic prostate cancer cell line PC‐3M‐1E8 and highly expressed in the low metastatic PC‐3M‐2B4. Furthermore, MMP‐9 and MMP‐2 activity were found to be significantly increased in LASS2/TMSG1 shRNA PC‐3M‐2B4 cells. Moreover, mouse xenografts of PC‐3M‐2B4 cells transfected with LASS/TMSG1 shRNA exhibited significantly increased average tumor size and weight compared to controls. Those with LASS/TMSG1 shRNA also had more LNM, suggesting loss of LASS2/TMSG1 induced tumor cell growth, proliferation, invasion, and metastasis likely as a result of loss of control in V‐ATPase activity.^96^

Furthermore, these modulatory proteins also may have a role in cancer therapy resistance. It has been demonstrated that the expression of LASS2/TMSG1 was significantly lower in doxorubicin resistant MCF‐7/ADR breast cancer cells than sensitive MCF‐7 cells. LASS2/TMSG1‐positive tumors had a positive correlation with disease‐free and overall survival. It was shown that overexpression of LASS2/TMSG1 increased chemosensitivity to a number of chemotherapeutic agents in drug resistant MCF‐7/ADR cells. Overexpression of LASS2/TMSG1 inhibited pHi recovery and significantly decreased MCF‐7/ADR cell migration due to the suppression of V‐ATPase function via LASS2/TMSG1 binding to subunit V_o_c.^26^ Furthermore, the downregulation of a positive regulator of V‐ATPase activity, TM9SF4, significantly inhibited tumor cell invasiveness and increased the cytotoxic effect of 5‐FU in colon cancer cells. This group hypothesized that in malignant cancer cells, TM9SF4 binds to the V_1_H subunit, leading to stabilization of the V‐ATPase complex and permanent activation of the enzyme.^25^

## 8 CONCLUSION

There is emerging evidence to support a role for V‐ATPase in cancer biology through impact on processes including cancer cell invasion, metastasis, and proliferation. Evidence also supports a potential link between V‐ATPase and conventional cancer therapy and that V‐ATPase may represent a direct anti‐cancer target. Future work is still required to understand whether the expression of individual subunits represents a casual or causal relationship with cancer progression. It is also important to uncover whether there are other functional consequences of V‐ATPase dysregulation in cancer and if more specific agents can be developed with more acceptable toxicity profiles.

## CONFLICT OF INTERESTS

None declared.
